# MiRNAs as Biomarkers of Myocardial Infarction: A Meta-Analysis

**DOI:** 10.1371/journal.pone.0088566

**Published:** 2014-02-12

**Authors:** Chao Cheng, Qiang Wang, Wenjie You, Manhua Chen, Jiahong Xia

**Affiliations:** 1 Cardiovascular Surgery, Union Hospital, Tongji Medical College, Huazhong University of Science and Technology, Wuhan, Hubei, China; 2 State Key Laboratory for Medical Genomics, Shanghai Institute of Hematology, Rui-Jin Hospital affiliated to School of Medicine, Shanghai Jiao Tong University, Shanghai, China; 3 Department of Respiratory and Critical Care Medicine, Union Hospital, Tongji Medical College, Huazhong University of Science and Technology, Wuhan, Hubei, China; 4 Department of Cardiovascular Medicine, The Central Hospital of Wuhan, Wuhan, China; 5 Department of Cardiovascular Surgery, The Central Hospital of Wuhan, Wuhan, China; University of Tampere, Finland

## Abstract

**Background:**

Recent studies have demonstrated that acute myocardial infarction induces a distinctive miRNA signature, suggesting that miRNAs may serve as diagnostic markers. Although many studies have investigated the use of miRNAs in the detection of cardiac injury, some had small sample sizes (<100 patients) or reported different results for the same miRNA. Here, the role of circulating miRNAs for use as biomarkers of myocardial infarction is summarized and analyzed.

**Methods and Results:**

Medline, SCI, Embase, and Cochrane databases were searched up to January 2013 for studies that evaluated associations between miRNAs and myocardial infarction. Relevant publications were identified by searching for combinations of “myocardial infarction,” “miRNAs,” and their synonyms. Methodological quality was scored using a standardized list of criteria, and diagnostic performance was assessed using estimates of test sensitivity and specificity. These values were summarized using summary receiver-operating characteristic curves. Nineteen studies met the inclusion criteria: 15 studies reported sensitivity, specificity, and AUC, but 4 studies did not. **Total miRNAs**: sensitivity: 0.78 (95%CI: 0.77–0.80; *P* = 0.0000); specificity: 0.82 (95%CI: 0.80–0.83; *P* = 0.0000). **miR-499**: sensitivity: 0.88 (95%CI:0.86–0.90; *P* = 0.0000); specificity: 0.87 (95%CI:0.84–0.90; *P* = 0.0000). **miR-1**: sensitivity: 0.63 (95%CI:0.59–0.66; *P* = 0.0000); specificity: 0.76 (95%CI:0.71–0.80; *P* = 0.0000). **miR-133a**: sensitivity: 0.89 (95%CI:0.83–0.94; *P* = 0.0047); specificity: 0.87 (95%CI:0.79–0.92; *P* = 0.0262). **miR-208b**: sensitivity: 0.78 (95%CI:0.76–0.81; *P* = 0.0581); specificity: 0.88 (95%CI:0.84–0.91; *P* = 0.0000). The correlation between miRNAs and other diagnostic biomarkers of myocardial infarction was obvious.

**Conclusion:**

MiRNAs, especially miR-499 and miR-133a, may be suitable for use as diagnostic biomarkers of myocardial infarction.

## Introduction

Coronary artery disease (CAD) and acute myocardial infarction (AMI) are the leading causes of death in developed and developing countries [1]. According to the American Heart Association, mortality caused by CAD in United States of America exceeded 400,000 in 2007, accounting for about 1 in every 6 deaths. Approximately every minute, someone in the U.S. dies from a heart attack [2]. AMI accounts for most of the mortality due to CAD. However, the mortality attributable to AMI in the U.S. has been declining, partly due to earlier recognition and effective revascularization therapy, including percutaneous coronary intervention (PCI) and coronary artery bypass surgery (CABG) [3]. Circulating biomarkers of myocardial damage, especially cardiac-specific troponin, have facilitated early diagnosis of AMI, maximizing the benefits of revascularization therapy. In AMI patients, troponin levels increase as early as 3.5 hours (h) after the onset of chest pain. However, due to the relative delay in the timing of the release of troponin, specific, sensitive biomarkers are in urgent demand and may further reduce AMI mortality [4] Most of the currently available biomarkers, the ones already in clinical use, are proteins and polypeptides [5]. Novel biomarkers, such as molecular and genetic biomarkers, are under investigation [6]. In recent years, miRNAs have been recognized as novel biomarkers because of their diverse but tissue- and cell-specific biological and pathological functions [7]–[10]. However, many issues are still unclear. Some studies evaluated the same miRNAs but reported markedly different results. For example, the sensitivity and specificity of miR-499 and miR-208b were lower in the study published by Corsten et al. than in other related studies [14].

In this review, recent information on biomarkers for AMI is summarized, focusing on the latest insights in the identification and potential use of miRNAs in the plasma and serum. The specificity and sensitivity of miRNAs were evaluated to assess the feasibility of using them as biomarkers of AMI.

## Materials and Methods

### Identification and selection of relevant studies

Sources of studies included the Medline, SCI, Embase, and Cochrane library databases. The databases were searched from inception to January 28, 2013 for relevant studies using the terms “myocardial infarction,” “heart infarction,” “heart injury,” and “cardiovascular infarction” in combination with “miRNAs” and “microRNA” and synonyms for all five terms. Potentially associated publications were assessed by checking their titles and abstracts and the most relevant publications were subjected to closer examination. The reference lists of the selected papers were also screened for articles that might have been missed in the initial search, and references cited in the identified articles were searched manually. A manual search of abstracts from 44th Annual Scientific Meeting of the European Society for Clinical Investigation, BAS/BSCR Poster Abstracts of the HEART, the Circulation Research, the ESC Congress 2012 was conducted.

The following criteria were used for the literature selection in the meta-analysis.

All eligible studies satisfied the following inclusion criteria:

1. miRNAs and myocardial infarctions were used in outcome analysis.

2. Sample size, sensitivity, specificity, AUC, and their 95% confidence intervals (CIs) or other information that might help assess the results was required.

3. Cohort studies and case-control studies were included.

4. There were no language restrictions.

5. A sample size of more than four subjects was required for each comparison group.

Accordingly, studies were excluded based on the following criteria:

1. Studies not conducted on humans.

2. Studies not mentioning myocardial infarction in the abstract.

3. Studies without comparison groups.

4. Studies that did not explicitly state that the control group consisted of human control subjects.

5. Studies those were designed or defined markedly differently from the selected papers.

6. Review articles, abstracts presented at conferences, editorials, commentaries, and studies without complete data.

### Data extraction and quality assessment

Variations in methodological quality of diagnostic studies may influence the results and conclusions of a systematic review. For this reason, the quality of each included study was assessed as follows. Two reviewers, Qiang Wang and Chao Cheng, independently scored the quality of the selected studies using a standardized set of criteria (study selection). These criteria have been used in previous reviews of diagnostic studies, and they were reported by Whiting et al. (10). Nine identical methodological criteria and three additional criteria were used in the present study (Appendix S1). Criteria were classified as “yes” (+), “no” (−), or “don’t know” (?). One point was given for each “yes,” and no points were given for each “no” and “don’t know.” All items were given equal weight, resulting in a maximum possible score of 12. Studies scoring over 7 were considered to have a low risk of bias. Data were extracted and entered into a database. The extraction was performed by two reviewers independently. In the case of conflicting evaluations, agreement was reached after discussion.

### Statistical analysis

The sensitivity, specificity, AUC, and diagnostic OR of each miRNA associated with the diagnostic value of myocardial infarction were estimated for each study. For detection of sample size bias, the sensitivity, specificity, AUC, and diagnostic OR and their 95% confidence intervals (CI) were plotted against the number of participants. The statistical heterogeneity was analyzed (χ2-based Q statistic test) and presented when I squared (I^2^) exceeded 50% or *P*<0.1. A random effect model (DerSimonian and Laird) was used for the meta-analysis in the case of significant heterogeneity (I^2^>50%) and a fixed effect model (Mantel-Haenszel) was used when the heterogeneity was not significant (I^2^<50%). The significance of the sensitivity, specificity, AUC, and diagnostic OR was determined using the last pooled values. Statistical analysis was undertaken using the Meta-disc 1.4 software.

## Results

**Figure 1:**
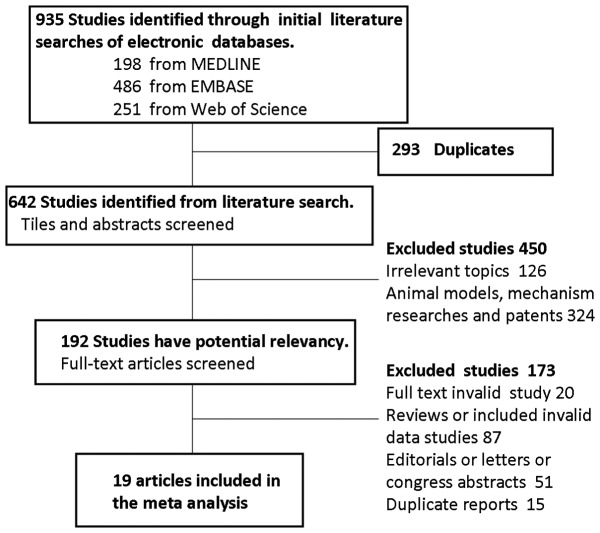
Flow diagram of the literature search and study selection.

A total of 935 potentially relevant abstracts were identified. After duplicates were removed, 642 unique abstracts remained. Abstracts and full-text articles were screened, and 19 publications seemed to meet all of the inclusion criteria and none of the exclusion criteria [11]–[26], [37]–[39] (Figure 1).

The 19 studies included 15 studies that reported the sensitivity, specificity, and AUC clearly [11]–[12], [14], [16]–[20], [22]–[25], [37]–[39] and 4 studies [13], [15], [21], [26] that did not provide this information. Among the 15 studies, 8 discussed miR-499 as a diagnostic biomarker of myocardial infarction [11], [14], [16], [22], [25], [37]–[39], 7 studies discussed miR-1 [12], [16]–[18], [37]–[39], 4 studies discussed miR-133a [16], [17], [24], [39], 6 papers discussed miR-208b [14], [16], [25], [37]–[39], and 5 studies evaluated another 9 types of miRNA [18]–[20], [23], [38]. Among the 19 included studies, 11 were set by hospital admission [11]–[13], [15], [16], [18], [19], [24], [37]–[39], 5 were based on CCU admission [14], [17], [20], [22], [26], and 3 were based on ED admission [21], [23], [25]. Data regarding changes in miRNAs over time were also recorded in the results (Table S1).

Almost all the studies included here, excepting only 1 [26], reported a correlation between miRNAs and other diagnostic biomarkers of myocardial infarction, such as cardiac troponin (cTnI and cTnT) or CKMB. Among 18 studies, 8 provided the line charts to demonstrate the correlations between these factors [11]–[14], [16], [17], [20], [25] (Table 1), 4 showed changes in the concentrations of miRNA and other biomarkers over time [15], [18], [19], [24] (Table 2), and 7 studies offered the AUC of miRNAs and the comparison biomarkers [21]–[23], [25], [37]–[39] (Table 3). In addition, 10 studies reported a correlation between miRNAs and cTnT [14], [16], [17], [20]–[22], [25], [37]–[39], 6 studies discussed cTnI [12], [15], [18], [19], [23], [24], 5 studies evaluated the relationship between miRNA and CKMB [11], [12], [13], [25], [38], and 2 studies described correlations between miRNA and other diagnostic methods [12], [14].

**Table 1:** Correlations between miRNAs and CKMB, cardiac troponin, and other diagnostic biomarkers.

Study	Comparison	miRNA	Comparison vs. miRNAs
Adachi, T. et al.	CKMB	miR-499	*P* = 0.0149
2010 [11]			Log_10_[miR-499] = 3.65+0.0044 * CKMB
Ai, J. et al.	QRS	miR-1	*P* = 0.0092
2010 [12]	ST segment	miR-1	*P* = 0.7772
	CKMB	miR-1	*P* = 0.6106
	cTnI	miR-1	*P* = 0.3326
Cheng, Y. et al.	CKMB	miR-1	*P*<0.05; r = 0.68
2010 [13]			CKMB = 97.89+101.84* miR-1
Corsten, M.F. et al.	CPK	miR-208b	*P* = 0.03; r = 0.4
2010 [14]	CPK	miR-499	*P* = 0.01; r = 0.41
	cTnT	miR-208b	*P* = 0.0005; r = 0.59
	cTnT	miR-499	*P*<0.0001; r = 0.69
Gidlof, O. et al.	cTnT	miR-208b	*P* = 0.01; r^2^ = 0.25
2011 [16]			
Kuwabara, Y. et al.	cTnT	miR-1	*P*<0.005; r^2^ = 0.1072
2011 [17]			Log[miR-1] = 0.1849×Log [cTnT] -38.618
	cTnT	miR-133a	*P*<0.0001; r^2^ = 0.3897
			Log[miR-133a] = 0.5009×Log[ cTnT] -35.51
Meder B. et al.	hsTnT	miR-30c miR-145	*P* = 0.0004; r = 0.713
2011 [20]			*P* = 0.0005; r = 0.710
Devaux, Y. et al.	hsTnT	miR-208b	ALL: *P* = 2*10^−17^; r = 0.36
2012 [25]			<3 h: *P* = 8*10^−7^; r = 0.38
			3–6 h: *P* = 0.002; r = 0.25
			6–12 h: *P* = 0.01; r = 0.26
	hsTnT	miR-499	ALL: *P*<10^−20^; r = 0.40
			<3 h: *P* = 4*10^−7^; r = 0.39
			3–6 h: *P* = 0.002; r = 0.25
			6–12 h: *P* = 0.0003; r = 0.37
	CK	miR-208b	*P* = 1*10^−17^; r = 0.37
	CK	miR-499	*P* = 1*10^−12^; r = 0.31
	cTnT	miR-208b	*P* = 7*10^−16^; r = 0.36
	cTnT	miR-499	*P* = 4*10^−10^; r = 0.29

Eight studies reporting the correlations between miRNAs and other biomarkers with line charts are displayed in the table.

**Table 2:** Results of AUC charts of correlations between miRNAs and cardiac troponin.

Study	Comparison				miRNAs				Comparison + miRNAs				Comparison vs miRNAs
	Type	Sensitivity	Specificity	AUC(95%Cl)	Type	Sensitivity	Specificity	AUC (95%Cl)	Type	Sensitivity	Specificity	AUC (95%Cl)	*P*
Oerlemans,	hsTnT	—	—	0.86	miR-1	ALL	ALL	0.75 (0.70–0.81)	hsTnT +miR-1			0.92 (0.90–0.95)	*P*<0.001
M.I.F.J. et al.				(0.82–0.91)	miR-208a	—	—	0.61 (0.54–9.67)	hsTnT +miR-208a			0.89 (0.85–0.93)	_
2012 [21]					miR-499			0.79 (0.74–0.84)	hsTnT +miR-499			0.92 (0.89–0.95)	*P*<0.001
					miR-21			0.76 (0.71–0.82)	hsTnT +miR-21			0.92 (0.89–0.95)	*P*<0.001
					miR-146a			0.68 (0.62–0.74)	hsTnT +miR-146a			0.90 (0.87–0.94)	_
					miR-1+ miR-499			0.89 (0.85–0.94)	hsTnT+miR-1+			0.94 (0.92–0.97)	*P*<0.001
					+miR-21				miR-499 +miR-21				
Olivieri, F. et al.	cTnT	—	—	1.00	miR-499-5p	1.00	1.00	1.00					
2012 [22]													
Wang, G.K. et	cTnI	<4 h:	_	0.987	miR-1	—	ALL	0.847 (0.751–0.943)					
al. 2010 [23]		0.85		(0.966–1.000)	miR-133a	—	—	0.867 (0.771–0.963)					
		>4 h:			miR-499	—		0.822 (0.717–0.927)					
		1.00			miR-208a	miR-208a		0.965 (0.92–1.00)					
		Total:				<4 h:1.00							
		0.909				>4 h:0.846							
						Total:0.909							
Devaux, Y. et	hsTnT	0.93	0.98	ALL: 0.97	miR-208b	0.79	1.00	ALL: 0.90	hsTnT +miR-499	0.95	0.98		ALL: *P* = 0.42
al.				<3 h: 0.94				<3 h: 0.83					<3 h: *P* = 0.26
2012 [25]				3–6 h: 0.98				3–6 h: 0.91					3–6 h: *P* = 0.48
				6–12 h: 0.99				6–12 h: 0.95					6–12 h: *P* = 0.28
					miR-499	0.95	1.00	ALL: 0.97					ALL: *P*<0.0001
								<3 h: 0.96					<3 h: *P*<0.0001
								3–6 h: 0.99					3–6 h: *P*<0.0001
								6–12 h: 0.99					6–12 h: *P* = 0.007
Gidlof, O. et al.	cTnT	0.95	0.95	0.95	miR-1	0.55	0.60	0.57					
2013 [37]					miR-208b	0.75	0.82	0.82					
					miR-499-5p	0.78	0.75	0.79					
Li, C. J. et al.	cTnT	0.62	0.98	0.800	miR-1	0.60	0.70	0.696 (0.593–0.799);					
2013 [38]				(0.714–0.887)	miR-134	0.55	0.75	0.657 (0.551–0.763);					
					miR-186	0.78	0.58	0.715 (0.614–0.817);					
					miR-208	0.78	0.76	0.778 (0.686–0.869);					
					miR-223	0.78	0.68	0.741 (0.645–0.838)					
	CKMB	0.62	0.75	0.683	miR-499	0.75	0.72	0.755 (0.662–0.849)					
				(0.579–0.786)									
Li, Y. Q. et al.	cTnT	0.95	1.00	0.9820	miR-1	0.78	0.85	0.8265					
2013 [39]				(0.9289–0.997				(0.7441–0.9088),					
				5)	miR-133a	0.88	0.96	0.9468					
								(0.9057–0.9879),					
					miR-208b	0.82	1.00	0.8899					
								(0.8259–0.9540),					
					miR-499	0.80	0.94	0.8841					
								(0.8187–0.9495),					

Seven studies reporting the correlations between miRNAs and cardiac troponin with sensitivity, specificity, and AUC values charts are displayed in the table.

**Table 3:** Results of time curve charts of correlations between miRNAs and cardiac troponin I biomarkers.

Study	Biomarkers	Time and fold change/max change							
D'Alessandra, Y. et al.		T0*	3 h	9 h	15 h	21 h	33 h	45 h	69 h
2010 [15]	cTnI	0.65	0.80	0.58	0.35	0.4	0.32	0.2	0.18
	miR-1	1.0	0.35	0.1	0.06	0.05	0.05	0.06	0.04
	miR-133a	1.0	0.18	0.05	0.04	0.03	0.02	0.03	0.02
	miR-133b	0.9	0.55	0.2	0.1	0.07	0.05	0.05	0.05
	miR-499-5p	0.58	0.65	0.88	0.5	0.3	0.1	0.01	0.01
	miR-122	0.8	0.4	0.4	0.3	0.45	0.4	0.2	0.4
	miR-375	1.0	0.3	0.2	0.19	0.21	0.1	0.2	0.4
Long, G. et al.			4 h	8 h	12 h	24 h	48 h	721h	1 w
2012 [18]	cTnI		0.8	0.8	0.55	0.6	0.3	0.2	0
	miR-1		0.2	1.0	0.35	0.1	0.3	0.3	0.2
	miR-126		0.4	1.0	0.7	0.2	0.8	0.3	0.1
Long, G. et al.			4 h	8 h	12 h	24 h	48 h	72 h	1 w
2012 [19]	cTnI		0.8	0.8	0.5	0.6	0.3	0.2	0
	miR-30a		0.15	1.0	0.15	0	0.1	0.1	0
	miR-195		0.1	1.0	0.15	0.1	0.05	0.05	0.05
Wang, R. et al.		Control		T0†		20 h		7 d	
2011 [24]	cTnI‡	1		15		18		1	
	miR-133§	0.4		0.15		0.2		0.35	
	miR-328§	0.2		−0.2		0.05		0.1	

Four studies reporting the fold changes/max changes or fold changes of miRNAs and cTnI with time are displayed in the table.

* T0 indicates 156±72 min after the onset of symptoms.

† T0 indicates 5.24±1.38 hours after AMI.

‡ Fold changes.

§ Log_2_ relative expression levels.

### Methodological quality assessment

Mean score of risk for bias of all 19 included studies was 7.789, ranging from 5.00 to 10.00. In the 19 studies, only two studies had scores below 7 [11], [12], seven studies had scores of 7 [14], [16], [18]–[20], [24], [26], and the scores of the remaining ten studies were all higher than 7 [13], [15], [17], [21]–[23], [25], [37]–[39]. Not all of the reference standard results or index test results were blinded. The highest score out of all sixteen studies was 10, and only two studies reached it [21], [25]. Five criteria, “acceptable delay between tests,” “partial verification avoided,” “differential verification avoided,” “incorporation avoided,” and “characteristics described,” were met by all studies included here. The lowest score was 5 and only one study showed it [11]. Representative spectra, acceptable reference standards, and defined inclusion criteria were the main causes of different scores among these studies (Table S2).

### Overall analysis

#### Total miRNA levels

**Figure 2:**
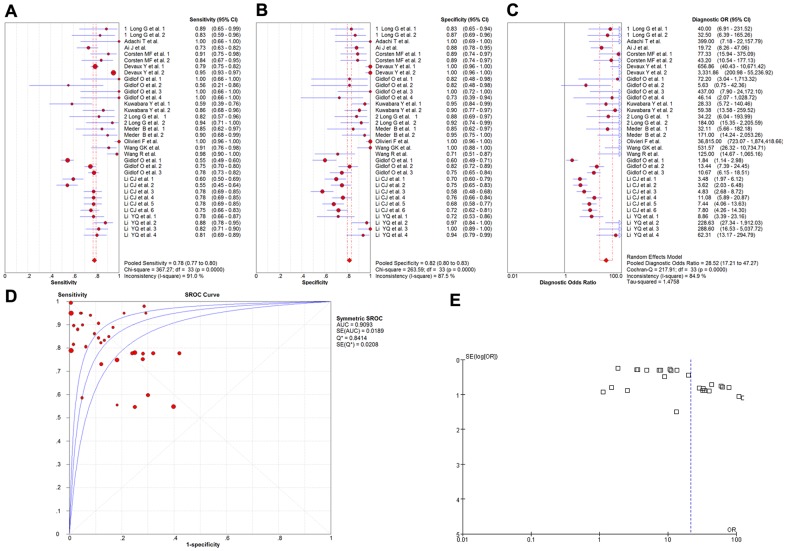
The sensitivity, specificity, diagnostic OR, SROC curve with AUC and funnel plot for total miRNA levels of 15 studies in the diagnosis of myocardial infarction. (A) Sensitivity. (B) Specificity. (C) Diagnostic OR. (D) SROC curve with AUC. (E) Funnel plot. Df, degree of freedom; OR, odds ratio; SROC, summary receiver operator characteristics; AUC, area under the curve; SE, standard error; Q*, Q index. Balls, estimated respectively the sensitivity, specificity, diagnostic OR, AUC; Bars, 95% confidence intervals (CIs); Width of diamonds, pooled CIs. The size of each ball is proportional to the weight of each study in the meta-analysis. The SROC show all values of AUC and the area between the upper left and lower right curves represent the CIs of AUC for total miRNA levels. Values that cross the borders are not shown in these figures. Boxes in the funnel plot indicated the studies included in this meta- analysis.

Some 15 studies covering 13 types of miRNA and involving a total of 2136 participants investigated the diagnostic values of miRNAs as the biomarkers of myocardial infarction [11]–[12], [14], [16]–[20], [22]–[25], [37]–[39], and a meta-analysis of the sensitivity, specificity, diagnostic OR, and SROC curve with AUC for miRNAs in the diagnosis of myocardial infarction was plotted. A random effect model was used for the meta-analysis due to significant heterogeneity (all I^2^>50%). The pooled sensitivity (Figure 2A), specificity (Figure 2B), and diagnostic OR (Figure 2C) with their 95% confidential intervals (95%CIs), *P*-values, and AUC values (Figure 2D) for total miRNA levels in the 15 studies were 0.78 (95%CI:0.77–0.80; *P* = 0.0000), 0.82 (95%CI:0.80–0.83; *P* = 0.0000), 28.52 (95%CI: 17.21–47.27; *P* = 0.0000), and 0.9093, respectively. In order to compare the differences and evaluate the sensitivity of the meta-analysis, a fixed-effect model was used to calculate all the variables. There were no differences among the results obtained using the random-effects model with respect to sensitivity or specificity. With respect to the diagnostic OR variable, the summary OR was 12.68 (95% CI: 11.00–14.62; *P* = 0.0000), which was not significantly different from the results obtained using the random-effect model. In addition, the funnel plot was used to assess publication bias, which suggested that publication bias probably had effect on summary estimates (Figure 2E).

#### miR-499

**Figure 3:**
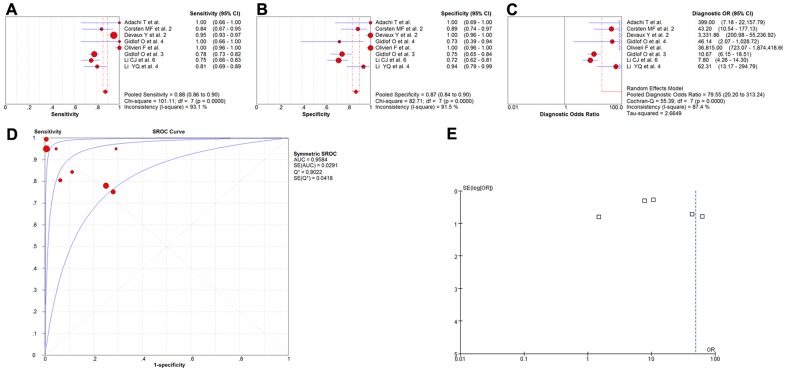
The sensitivity, specificity, diagnostic OR, SROC curve with AUC and funnel plot for miR-499 levels of 8 studies in the diagnosis of myocardial infarction. (A) Sensitivity. (B) Specificity. (C) Diagnostic OR. (D) SROC curve with AUC. (E) Funnel plot. Df, degree of freedom; OR, odds ratio; SROC, summary receiver operator characteristics; AUC, area under the curve; SE, standard error; Q*, Q index. Balls, estimated respectively the sensitivity, specificity, diagnostic OR, AUC; Bars, 95% confidence intervals (CIs); Width of diamonds, pooled CIs. The size of each ball is proportional to the weight of each study in the meta-analysis. The SROC show all values of AUC and the area between the upper left and lower right curves represent the CIs of AUC for total miRNA levels. Values that cross the borders are not shown in these figures. Boxes in the funnel plot indicated the studies included in this meta- analysis.

Eight studies covering 1634 participants evaluated the diagnostic value of miR-499 as a biomarker of myocardial infarction [11], [14], [16], [22], [25], [37]–[39]. The meta-analysis of the sensitivity, specificity, diagnostic OR and SROC curve was plotted with AUC for miR-499 in the diagnosis of myocardial infarction. A random effect model was used for the meta-analysis because of the heterogeneity (all I^2^>50%). The pooled sensitivity (Figure 3A), specificity (Figure 3B), and diagnostic OR (Figure 3C) with their 95% confidential intervals (95%CIs) and *P*-values and the AUC value (Figure 3D) of the miR-499 in the 8 studies were 0.88 (95%CI: 0.86–0.90; *P* = 0.0000), 0.87 (95%CI: 0.84–0.90; *P* = 0.0000), 79.55 (95%CI: 20.20–313.24; *P* = 0.0000), and 0.9584, respectively. In order to compare results of different studies and evaluate the sensitivity of the meta-analysis, a fixed-effect model was used to calculate all variables. No significant differences were observed among the results obtained from the random-effect model with respect to sensitivity or specificity. In the diagnostic OR variable, the summary OR was 27.53 (95% CI: 20.42–37.12; *P* = 0.0000), which was not significantly different from the results obtained using the random-effect model. In addition, the funnel plot was used to assess publication bias, which suggested that publication bias probably had significant effect on summary estimates (Figure 3E).

#### miR-1

**Figure 4:**
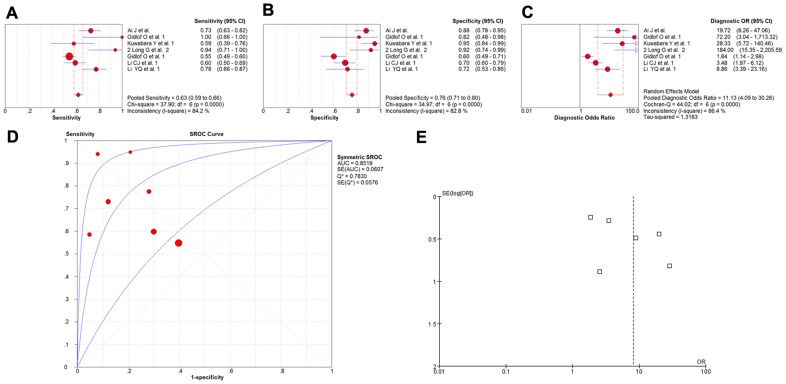
The sensitivity, specificity, diagnostic OR, SROC curve with AUC and funnel plot for miR-1 levels of 7 studies in the diagnosis of myocardial infarction. (A) Sensitivity. (B) Specificity. (C) Diagnostic OR. (D) SROC curve with AUC. (E) Funnel plot. Df, degree of freedom; OR, odds ratio; SROC, summary receiver operator characteristics; AUC, area under the curve; SE, standard error; Q*, Q index. Balls, estimated respectively the sensitivity, specificity, diagnostic OR, AUC; Bars, 95% confidence intervals (CIs); Width of diamonds, pooled CIs. The size of each ball is proportional to the weight of each study in the meta-analysis. The SROC show all values of AUC and the area between the upper left and lower right curves represent the CIs of AUC for total miRNA levels. Values that cross the borders are not shown in these figures. Boxes in the funnel plot indicated the studies included in this meta- analysis.

Seven studies involving of 1031 participants investigated the diagnostic values of miR-1 as the biomarkers for myocardial infarction [12], [16]–[18], [37]–[39], and the meta-analysis of the sensitivity, specificity, diagnostic OR and the SROC curve with AUC for miR-1 in the diagnosis of myocardial infarction was plotted. A random effect model was used for the meta-analysis because of the heterogeneity (all I^2^>50%). The pooled sensitivity (Figure 4A), specificity (Figure 4B), and diagnostic OR (Figure 4C) with their 95% confidential intervals (95%CIs) and *P*-values and the AUC value (Figure 4D) of the miR-1 in the 7 studies were 0.63 (95%CI:0.59–0.66; *P* = 0.0000), 0.76 (95%CI:0.71–0.80; *P* = 0.0000), 11.13 (95%CI: 4.09–30.26; *P* = 0.0000), and 0.8519, respectively. In order to compare results of different studies and evaluate the sensitivity of the meta-analysis, a fixed-effect model was used to calculate all variables. No significant differences were observed among the results obtained from the random-effect model with respect to sensitivity or specificity. In the diagnostic OR variable, the summary OR was4.77 (95% CI: 3.65–6.24; *P* = 0.0000), which was not significantly different from the results obtained using the random-effect model. In addition, the funnel plot was used to assess publication bias, which suggested that publication bias probably had little effect on summary estimates (Figure 4E).

#### miR-133a

**Figure 5:**
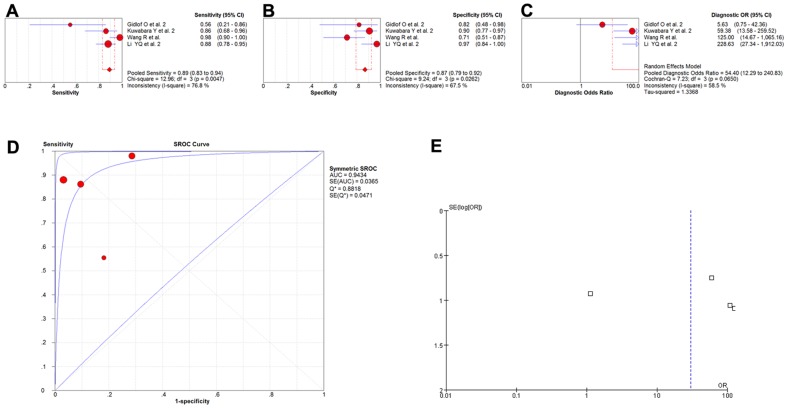
The sensitivity, specificity, diagnostic OR, SROC curve with AUC and funnel plot for miR-133a levels of 4 studies in the diagnosis of myocardial infarction. (A) Sensitivity. (B) Specificity. (C) Diagnostic OR. (D) SROC curve with AUC. (E) Funnel plot. Df, degree of freedom; OR, odds ratio; SROC, summary receiver operator characteristics; AUC, area under the curve; SE, standard error; Q*, Q index. Balls, estimated respectively the sensitivity, specificity, diagnostic OR, AUC; Bars, 95% confidence intervals (CIs); Width of diamonds, pooled CIs. The size of each ball is proportional to the weight of each study in the meta-analysis. The SROC show all values of AUC and the area between the upper left and lower right curves represent the CIs of AUC for total miRNA levels. Values that cross the borders are not shown in these figures. Boxes in the funnel plot indicated the studies included in this meta- analysis.

Four studies involving 285 participants investigated the diagnostic values of miR-133a as the biomarkers for myocardial infarction [16], [17], [24], [39], and the meta-analysis of the sensitivity, specificity, diagnostic OR, and SROC curve with AUC for miR-133a was plotted for the diagnosis of myocardial infarction. A random effect model was used for the meta-analysis due to significant heterogeneity (all I^2^>50%). The pooled sensitivity (Figure 5A), specificity (Figure 5B), and diagnostic OR (Figure 5C), with their 95% confidential intervals (95%CIs) and *P*-values, and the AUC value (Figure 5D) of miR-133a were 0.89 (95%CI: 0.83–0.94; *P* = 0.0047), 0.87 (95%CI:0.79–0.92; *P* = 0.0262), 54.40 (95%CI: 12.29–240.83; *P* = 0.0650), and 0.9434, respectively. In order to compare the differences in results and evaluate the sensitivity of the meta-analysis, a fixed-effect model was used to calculate all the variables. No differences were observed among results obtained using the random-effect model with respect to sensitivity or specificity. With respect to the diagnostic OR variable, the summary OR was 57.92 (95% CI: 23.76–141.18; *P* = 0.0650), which was not significantly different from the results obtained using the random-effect model. In addition, the funnel plot was used to assess publication bias, which suggested that publication bias probably had effect on summary estimates (Figure 5E).

#### miR-208b

**Figure 6:**
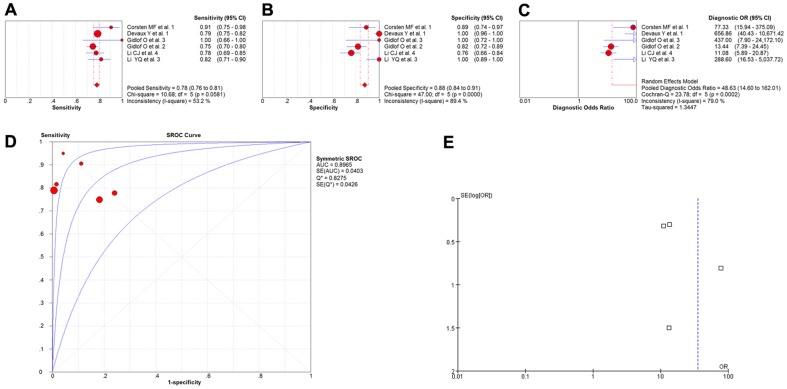
The sensitivity, specificity, diagnostic OR, SROC curve with AUC and funnel plot for miR-208b levels of 6 studies in the diagnosis of myocardial infarction. (A) Sensitivity. (B) Specificity. (C) Diagnostic OR. (D) SROC curve with AUC. (E) Funnel plot. Df, degree of freedom; OR, odds ratio; SROC, summary receiver operator characteristics; AUC, area under the curve; SE, standard error; Q*, Q index. Balls, estimated respectively the sensitivity, specificity, diagnostic OR, AUC; Bars, 95% confidence intervals (CIs); Width of diamonds, pooled CIs. The size of each ball is proportional to the weight of each study in the meta-analysis. The SROC show all values of AUC and the area between the upper left and lower right curves represent the CIs of AUC for total miRNA levels. Values that cross the borders are not shown in these figures. Boxes in the funnel plot indicated the studies included in this meta- analysis.

Six studies involving 1424 participants evaluated the diagnostic value of miR-208b as a biomarker of myocardial infarction [14], [16], [25], [37]–[39], and a meta-analysis of the sensitivity, specificity, diagnostic OR, and SROC curve with AUC for miR-208b in the diagnosis of myocardial infarction was plotted. A random effect model was used for the meta-analysis due to significant heterogeneity (all I^2^>50%). The pooled sensitivity (Figure 6A), specificity (Figure 6B), and diagnostic OR (Figure 6C), with their 95% confidential intervals (95%CIs) and *P*-values, and the AUC value (Figure 6D) of miR-208b were 0.78 (95%CI: 0.76–0.81; *P* = 0.0581), 0.88 (95%CI:0.84–0.91; *P* = 0.0000), 48.63 (95%CI: 14.60–162.01; *P* = 0.0002), and 0.8965, respectively. In order to compare the differences in results and evaluate the sensitivity of the meta-analysis, a fixed-effect model was used to calculate all the variables. No differences were observed among results obtained using the random-effect model with respect to sensitivity or specificity. With respect to the diagnostic OR variable, the summary OR was 26.66 (95% CI: 18.47–38.49; *P* = 0.0002), which was not significantly different from the results obtained using the random-effect model. In addition, the funnel plot was used to assess publication bias, which suggested that publication bias probably had some effect on summary estimates (Figure 6E).

## Discussion

### Sensitivity and specificity in miRNAs

In the present study, the diagnostic value of miRNAs as biomarkers of myocardial infarction was evaluated based on observations made in relevant previous studies. Sensitivity and specificity are basic standards used to estimate the suitability of one diagnostic method. The diagnostic OR and SROC curve (AUC) values can be used to describe the characteristics of index test and its suitability as a diagnostic method. The results described above show that miRNAs are suitable for use as diagnostic biomarkers of myocardial infarction. Total miRNA levels: sensitivity: 0.78 (95%CI: 0.77–0.80; *P* = 0.0000); specificity: 0.82 (95%CI: 0.80–0.83; *P* = 0.0000); diagnostic OR: 28.52 (95%CI: 17.21–47.27; *P* = 0.0000); AUC: 0.9093. Four miRNAs that had been evaluated repeatedly were chosen for subgroup analysis: miR-499, miR-1, miR-133a, and miR-208b. MiR-499 showed satisfactory sensitivity and specificity values (sensitivity: 0.88 (95%CI: 0.86–0.90; *P* = 0.0000); specificity: 0.87 (95%CI: 0.84–0.90; *P* = 0.0000)). The specificity of miR-208b was better than its sensitivity (sensitivity: 0.78 (95%CI: 0.76–0.81; *P* = 0.0581); specificity: 0.88 (95%CI: 0.84–0.91; *P* = 0.0000)), and the sensitivity of miR-133a was better than its specificity (sensitivity: 0.89 (95%CI: 0.83–0.94; *P* = 0.0047); specificity: 0.87 (95%CI: 0.79–0.92; *P* = 0.0262)). MiR-1 (sensitivity: 0.63 (95%CI: 0.59–0.66; *P* = 0.0000); specificity: 0.76 (95%CI: 0.71–0.80; *P* = 0.0000)) were disappointing, because of their low sensitivity and specificity values. Almost all the heterogeneities in this study were very pronounced. This was because of factors such as region, age, race, and other patient characteristics.

Among all miRNAs investigated in this study, miR-499 was found to be most significantly associated with myocardial infarction. The results given above show not only the sensitivity and specificity of miR-499 to be satisfied but also highlight certain other characteristics. In the study published by Devaux et al., miR-499 levels were evaluated within hours of the onset of symptoms, which is helpful for early diagnosis [25]. In the same study, the peak fold change in the concentration of miR-499 was found to reach 3*10∧5, which made detection of miR-499 relatively easy. Another factor found to affect the detection of miR-499 was the duration of elevated concentration. The longest duration reported in the studies evaluated here was 5 days, in the study published by D'Alessandra1 et al. [15]. However, this figure may not be accurate because of the limits of the detection techniques and other factors. MiR-1, miR-133a, and miR-208b have also been evaluated in many studies. In the study made public by Wang et al., miR-208 was found to be more sensitive and specific than miR-1, miR-133a, or miR-208b [23]. However, in the present study, miR-499 was found to be its superior.

Other miRNAs in the studies included here have their own characteristics. In a study by Vogel et al., the reported miRNAs changed greatly not only early during myocardial infarction, but also during mild infarction [26]. In a study by D'Alessandra1 et al., miR-122 and miR-375 showed obvious degradation, the duration of which could last 30 days after the infarction [15]. People who must get treatment immediately after myocardial infarction often miss the best time to be treated. This is especially critical in China because of the social and economic environment. The long duration could help to solve this problem.

### Correlations between miRNAs and cTnT, cTnI, and CKMB

CKMB and cardiac troponin have been widespread used in the diagnosis of myocardial infarction. Correlations between miRNAs and other diagnostic biomarker of myocardial infarction are presented in the current study. Although some studies have used lines chart to show these relationships, some have used the AUC value and others showed their changes over time. In either case, obvious correlations are easily discovered. Ten studies reported the correlation between miRNAs and cTnT. Six studies discussed cTnI. Five studies showed the relationship between miRNAs and CKMB. The results shown in these line charts indicate that some miRNAs, like miR-1, miR122, miR375, and miR-328, changed concentration earlier than cardiac troponin I, and levels of change of some miRNAs were more obvious than others [15], [24]. In studies that reported AUC values, the AUC values of single miRNAs, cTnT/I, and CKMB were not high enough to meet the standards of diagnostic biomarkers. Some studies reported the effects of miRNAs combined with other biomarkers and the combined effects to be satisfactory. Although some miRNAs, like miR-1 and miR-21, may not be suitable biomarkers, they could be used to diagnose myocardial infarction when combined with other biomarkers [21].

The correlations between miR-499 and CKMB, cTnT, hs-cTnT, cTnI, and CPK merit concern. The line charts of the related studies showed the obvious correlations between miR-499 and CKMB, cTnT, hs-cTnT, and CPK [11], [14], [25]. In three studies, the AUC values of miR-499, cTnT, hs-cTnT, and their combinations were reported [21], [22], [25]. In the research published by Olivieri et al., the AUC values miR-499 and cTnT were all 1.00 [22]. In the study published by Martinus et al., the AUC value of single hscTnT or miR-499 was lower than 0.90, but the AUC value of the combination of the two was 0.92 [21]. However, the reverse was reported in a study by Devaux et al., in which the AUC value of either miR-499 or hs-cTnT alone was higher than that of the two combined [25]. More studies are needed to explain this discrepancy.

### Feasibility of miRNAs as diagnostic biomarkers of myocardial infarction

MiRNA profiles may be useful in the early detection of AMI. Early detection biomarkers may indicate the onset of a disease and often play a role in the disease [27], [28]. cTNT and cTNI can be released into the serum during necrosis occurs in the process of AMI. However, the release of miRNA can be affected by any form of cellular stress, such as anoxia, lactic acidosis, or cellular edema. In AMI, these occur earlier than necrosis. Although several methods of RNA detection have been developed, both simple and complex methods tend to be expensive and time-consuming [29]. There are some promising methods, but they require improvement before they can be used in clinical settings or hospitals. For instance, bioluminescence miRNA detection technology, solution-phase bioluminescence methods, and high-throughput miRNA sequencing, also called “next generation sequencing,” have shown some promise [30]–[34]. New research suggests that continuous improvement may be possible. Wang et al. used a programmable oligonucleotide probe to generate a target-specific signature signal that can quantify subpicomolar levels of cancer-associated miRNAs and distinguish single-nucleotide differences between miRNA family members without the need for labels or miRNA amplification [35]. Zhao et al. reported a new isothermal reaction, which they used to simultaneously amplify and detect RNA. This method was based on cleavage using a DNAzyme and signal amplification, which cannot be contaminated by genomic DNA and is suitable for the detection of both mRNA and microRNA targets. The method showed high specificity and sensitivity [36]. Because of the emergency of miRNA biomarkers for disease, more and more attention has been drawn to miRNA detection technology. Instruments that can detect miRNA accurately, rapidly, conveniently, and inexpensively may be used in hospitals around the world in the near future.

There are some shortcomings in the research for warranting a meta-analysis. The sample sizes the cases and controls are often poorly matched and there is a lack of standardization. For example, different normalization procedures have been used without taking effects of medication into consideration. Another limitation of our study is that it is based on a limited number of articles. There are two reasons for it. For one, in some article the patients included are ACS which contains AMI, the specific number of AMI is unclear in the groups. We once asked the author for the data, however we failed to get the information. For another, although some studies detected the variation of miRNA in the AMI, the data did not meet our demand because of the different purposes of research. To make sure the reliability of our study, we excluded several articles [40]-[42]. Therefore, more researches are needed.

## Conclusion

It is possible that the miRNAs, particularly miR-499 and miR-133a, may be suitable for use as diagnostic biomarkers of myocardial infarction. MiR-208b suggests that it may also be usable, but it requires further evaluation. Other miRNAs may also be available, but more clinical studies are required to prove this.

## Supporting Information

Table S1 **All characteristics of all studies on the use of miRNAs as diagnostic biomarkers of myocardial infarction.**
(PDF)

Table S2 **QUADAS studies of diagnostic test accuracy.**
(PDF)

Appendix S1 **Methodology checklist: modified QUADAS for measuring diagnostic test accuracy.**
(DOC)
