# Supplementary material for: MiRNAs as Biomarkers of Myocardial Infarction: A Meta-Analysis
Source: PLoS One. 2014 Feb 12;9(2):e88566. doi: 10.1371/journal.pone.0088566 (PMC3922900; doi:10.1371/journal.pone.0088566)
Supplement: Table S1 — All characteristics of all studies on the use of miRNAs as diagnostic biomarkers of myocardial infarction. (PDF) [file pone.0088566.s001.pdf]

Table S1. All characteristics of all studies on the use of miRNAs as diagnostic biomarkers of myocardial infarction

Study (author, year, reference)	MiRNA biomarker (fold change)	Study design (location and setting)	Study population (number, age, sex: m/f)		Source	AMI diagnosis	MiRNA (1) Isolation (2) Detection (3) Normalization	Multivariate analysis	Quality score	Time of blood sampling	Base level (fold)	Time of measurable increase	Peak level (fold)	Peak time	Duration	Sensitivity	Specificity	AUC
			Case	Control														
Adachi, T. et al. 2010 [11]	miR-499 ↑	Japan<br>Hospital admission	9 patients with AMI<br>Age: 66.8±9.28<br>Sex (m/f): 6/3	10 healthy adults<br>Age: 41.5±8.0<br>Sex (m/f): 5/5	Plasma	None	(1) mirVana PARIS Kit<br>(2) qPCR, TaqMan<br>(3) Small nuclear RNA U6.	No	5	<48 h	—	<48 h	—	6–12 h	Before discharge	1.00	1.00	—
Ai, J. et al. 2010 [12]	miR-1 ↑	China<br>Hospital admission	93 patients with AMI<br>Age: 58.2±10.2<br>Sex (m/f): 67/26	66 healthy adults<br>Age: 55.1±9.6<br>Sex (m/f): 39/27	Plasma	Ischemic symptoms; increased cTnI and CKMB; pathological Q wave; ST-segment elevation or depression	(1) mirVana miRNA isolation kit<br>(2) qPCR<br>(3) Small nuclear RNA U6.	No	6	—	—	—	—	—	—	0.73	0.88	0.774
Cheng, Y. et al. 2010 [13]	miR-1 ↑	China<br>Hospital admission	31 patients with AMI<br>Age: 57±0.1<br>Sex (m/f): 18/13	20 non AMI with at least one-vessel disease patients<br>Age: 62±13<br>Sex (m/f): 20/12	Serum	Chest pain characteristic of myocardial ischemia lasting 30 min or more; ST segment elevation within 6 h of chest pain of at least 0.1 mV in at least two leads of the ECG; confirmation of the diagnosis of AMI by elevated by CK-MB isoenzyme in serum, which was at least twice the normal range	(1) mirVana miRNA isolation kit<br>(2) qPCR<br>(3) miR-1	No	8	8.5 ± 3.82 h	1	—	100	6 h	7 days	—	—	—
Corsten, M.F. et al. 2010 [14]	miR-208b ↑<br>miR-499 ↑	The Netherlands; Belgium; Luxembourg<br>CCU admission	32 patients with AMI<br>Age: 62±13<br>Sex (m/f): 20/12	36 non AMI with atypical chest pain patients<br>Age: 62±13<br>Sex (m/f): 23/13	Plasma	Chest pain <12 h with significant ST elevation and increase; Ck and Tnl to greater than 2-fold the upper limit of normal	(1) mirVana PARIS kit<br>(2) qPCR, miScript kit<br>(3) 3 *C. elegans* spiked-in miRNAs	No	7	8.5 ± 3.82 h<br><12 h of onset of chest pain	1<br>1<br>1	—<br><12 h<br><12 h	100<br>1600<br>100	6 h<br><12 h<br><12 h	7 days<br>—<br>—	—<br>0.91<br>0.85	—<br>0.89<br>0.89	—<br>0.944<br>0.918
D'Alessandra, Y. et al. 2010 [15]	miR-1 ↑<br>miR-133a ↑<br>miR-133b ↑<br>miR-499-5p ↑<br>miR-122 ↓<br>miR-375 ↓	Italy<br>Hospital admission	33 STEMI patients<br>Group1<br>25 patients<br>Age: 57.9±8.6<br>Sex (m/f): 24/1<br>Group2<br>8 patients<br>Age: 61.9±9<br>Sex (m/f): 7/1	17 healthy people<br>Age: 46.1±13.9<br>Sex (m/f): 13/4	Plasma	cTnl; acute anterior STEMI undergoing PCI; time symptom-to-balloon > 2 h and < 12 h; EF post PCI <= 45%; TIMI flow > 2 post PCI	(1) TRIzol LS reagent, mirVana PARIS<br>(2) qPCR, TaqMan<br>(3) miR-17-5p	Yes (age)	9	Group1: 25 patients 517 ± 309 min (day 0), day 5, day 30<br>Group2: 8 patients 156 ± 72 min after the onset of symptoms (T0) and again at 3, 9, 15, 21, 33, 45, and 69 h	Group 1<br>1<br>1<br>1<br>1<br>1<br>1<br>Group 2<br>ALL<br>—	Group 1<br>517 ± 309 min<br>517 ± 309 min<br>517 ± 309 min<br>517 ± 309 min<br>517 ± 309 min<br>517 ± 309 min<br>Group 2<br><156 ± 72 min<br><156 ± 72 min<br><156 ± 72 min<br><156 ± 72 min<br>—<br>—	Group 1<br>15<br>140<br>60<br>100<br>—<br>—<br>Group 2<br>48.3±17.4<br>5426±3047<br>312.2±182.6<br>299.1±106.4<br>—<br>—	Group 1<br>ALL<br>—<br>Group 2<br><156 ± 72 min<br><156 ± 72 min<br><156 ± 72 min<br>9 h<br>—<br>—	Group 1 and Group 2<br><5 days<br><5 days<br><5 days<br><30 days<br><5 days	ALL<br>—	ALL<br>—	ALL<br>—
Gidlof, O. et al. 2011 [16]	miR-1 ↑<br>miR-133a ↑<br>miR-208b ↑<br>miR-499-5p↑	Sweden<br>Hospital admission	25 STEMI patients<br>Age: 64.56±2.70<br>Sex (m/f): 20/5	11 healthy people<br>Age: 65.09±3.51<br>Sex (m/f): 7/4	Plasma	Patients undergoing primary percutaneous coronary intervention at Skane University Hospital Lund due to an STEMI were eligible for inclusion	(1) TRIzol LS reagent, miRNeasy mini kit<br>(2) qPCR<br>(3) Ct values of the miRNAs of interest were normalized against the arbitrary value of 40	No	7	First within 24 h (<12 h, n=9 and >12 h, n=16) of the onset of symptoms, second within 48 h, and third within 72 h.	ALL<br>—	<12 h<br><12 h<br><12 h<br><12 h	300<br>70<br>3000<br>250	<12 h<br><12 h<br><12 h<br><12 h	>72 h<br>>72 h<br>>72 h<br>>72 h	<12 h:Patients, n = 9 controls, n = 11<br>1.00<br>0.556<br>1.00<br>1.00	<12 h:Patients, n = 9 controls, n = 11<br>0.82<br>0.82<br>1.00<br>0.73	<12 h:Patients, n = 9 controls, n =11<br>0.98<br>0.86<br>1.00<br>0.99
Kuwabara, Y. et al. 2011 [17]	miR-1↑<br>miR-133a↑	Japan<br>CCU admission	29 ACS (21 MI and 8 UAP)<br>Age: 69.7±2.4<br>Sex (m/f): 23/6	42 non ACS people<br>Age: 69.2±2.2<br>Sex (m/f): 24/18	Serum	CK-MB; cTnT; pathological Q wave; ST-segment elevation or depression; chest pain	(1) TRIzol LS reagent<br>(2) qPCR, TaqMan<br>(3) Small nuclear RNA U6	No	8	miR-1<br><3 h, 3–9 h, 9–15 h, 15–21 h, >21 h<br>miR-133a<br><3 h, 3–9 h, 9–15 h, 15–21 h, 21–27 h, 27–33 h, >33 h	ALL<br>—	<3 h<br><3 h	ALL<br>—	<3 h<br><3 h	<15 h<br><27 h	0.60<br>0.87	0.96<br>0.91	0.777<br>0.932
Long, G. et al. 2012 [18]	miR-1↑<br>miR-126 ↓	China<br>Hospital admission	17 patients with AMI<br>Age: 53±12.5<br>Sex (m/f): 13/4	25 healthy adults<br>Age: 51±12.3<br>Sex (m/f): 18/7	Plasma	CK-MB; cTnl; pathological Q wave; ST-segment elevation or depression; ischemic symptoms	(1) TRIzol LS reagent<br>(2) qPCR<br>(3) Small nuclear RNA U6	No	7	4 h (± 30 min), 8 h (± 30 min), 12 h (± 30 min), 24 h (± 30 min), 48 h (± 30 min), 72 h (± 30 min), and 1 w (± 60 min) after the onset of symptoms	1<br>1	<4 h<br>4 h: 3.63±0.94<br>8 h: 15.87±4.5<br>12 h: 5.9±1.03<br>24 h: 2.37±0.50<br>48 h: 4.9±1.34<br>72 h: 4.3±1.2<br>1 w: 2.38±0.58<br>miR-126 levels in AMI patients were lower than in healthy controls at< 4 h	15.87 ± 4.5<br>—	8 h<br>—	1 w<br>1 w	4 h: 0.93, 8 h: 0.93, 12 h: 0.94, 24 h: 0.93, 48 h: 0.93, 72 h: 0. 90<br>4 h: 0.81, 8 h: 0.81, 12 h: 0.81, 24 h: 0.81, 48 h: 0.81, 72 h: 0.81 miRNA+: 14; miRNA-: 3	4 h: 0.90, 8 h: 0.90, 12 h: 0.93, 24 h: 0.90, 48 h: 0.90, 72 h: 0.90<br>4 h: 0.78, 8h : 0.84, 12 h: 0.88, 24 h: 0.77, 48 h: 0.80, 72 h: 0.70 miRNA+: 3; miRNA-: 22	4 h: 0.92, 8 h: 0.90, 12 h: 0.94, 24 h: 0.92, 48 h: 0.96, 72 h: 0.90<br>4 h: 0.86, 8 h: 0.87, 12 h: 0.88, 24 h: 0.87, 48 h: 0.84, 72 h: 0.83
Long, G. et al. 2012 [19]	miR-30a↑<br>miR-195↑	China<br>Hospital admission	18 patients with AMI<br>Age: 55± 11.4<br>Sex (m/f): 13/5	30 healthy adults<br>Age: 50± 12.3<br>Sex (m/f): 17/13	Plasma	CK-MB; cTnl; pathological Q wave; ST-segment elevation or depression; ischemic symptoms	(1) TRIzol LS reagent<br>(2) qPCR<br>(3) Small nuclear RNA U6	No	7	4 h (± 30 min), 8 h (± 30 min), 12 h (± 30 min), 24 h (± 30 min), 48 h (± 30 min), 72 h (± 30 min), and 1 w (± 60 min) after the onset of symptoms	1<br>1	<4 h<br>4 h: 1.4±60.37<br>8 h: 10.48 ±62.75<br>12 h: 1.45± 60.34<br>4–8 h<br>8 h: 10.2 ± 61.61<br>12 h: 1.4 ± 60.3	10.48 ± 2.75<br>10.2 ± 1.61	8 h<br>8 h	1 w<br>1 w	4 h: 0.88, 8 h: 0.88, 12 h: 0.82<br>8 h: 0.82, 12 h: 0.86,	4 h: 0.83, 8 h: 0.80, 12 h: 0.80<br>8 h: 0.88, 12 h: 0.80	4 h: 0.88, 8 h: 0.89, 12 h: 0.87<br>8 h: 0.89, 12 h: 0.88
Meder B. et al. 2011 [20]	miR-1291 ↓<br>miR-663b ↓<br>miR-30c↑<br>miR-145↑	German<br>CCU admission	20 STEMI patients<br>Age: 59.3±14<br>Sex (m/f): 16/4	20 healthy adults<br>Age: 63.3±14.8<br>Sex (m/f): 14/6	Whole blood	ESC/AHA redefined guidelines	(1) miRNeasy Mini Kit<br>(2)qPCR, Geniom Biochip miRNA<br>(3) Small nuclear RNA RNU6B-2	No	7	3.0 ± 2.3 h after the onset of symptoms and at 3 d and 4 d	ALL<br>—	ALL<br>—	ALL<br>—	ALL<br>—	ALL<br>—	0.85<br>0.90<br>—	0.85<br>0.95<br>—	0.91<br>0.94<br>—
Oerlemans, M. I. F. J. et al. 2012 [21]	miR-1 ↑<br>miR-208a ↑<br>miR-499↑<br>miR-21 ↑<br>miR-146a↑	The Netherlands<br>ED admission	106 ACS patients (82 with NSTEMI, 24 with UA)<br>Age: 68.7±12.6<br>Sex (m/f): 70/36	226 non-ACS patients with CHD<br>Age: 60.2±14.3<br>Sex (m/f): 120/106	Serum	Patients were enrolled at the ED of the Meander Medical Center (Amersfoort, the Netherlands) between May 2007 and November 2007 STEMI—these patients underwent immediate PCI—or refusal to provide informed consent were the only exclusion criteria	(1) TRIzol LS Reagent<br>(2) qPCR, TaqMan<br>(3) Small nuclear RNA U6	Yes	10	<3 h	NSTEM<br>1<br>1<br>1<br>1<br>1	NSTEM<br><3 h<br><3 h<br><3 h<br><3 h<br><3 h	ACS / NSTEM<br>10 / 8<br>2.3 / 2.3<br>23 / 20<br>18.9 / 18.9<br>11.7 / 13	ALL<br>—	ALL<br>—	ALL<br>—	ALL<br>—	ACS<br>0.75<br>0.61<br>0.79<br>0.76<br>0.68<br>miR-1+miR-499+miR-21: 0.89
Olivieri, F. et al. 2012 [22]	miR-499-5p↑	Italy<br>CCU admission	92 patients with NSTEMI (74% CHF)<br>Age: 82.6±6.9<br>Sex (m/f): 39/53	99 age matched healthy subjects<br>Age: 79.5±5.4<br>Sex (m/f): 40/59	Plasma	Clinical symptoms potentially correlated to AMI, typical chest pain or sudden onset of dyspnea; typical ECG alterations, either transient or persistent ST-segment elevation or depression>1 mm, persistent and definite T-wave inversion, including the pseudo normalization of previously negative T waves in ≥2 contiguous leads and new onset of complete left ventricular block branch; significant rise and subsequent fall in cTnT within the first 48–72 h	(1) mirVana PARIS<br>(2) qPCR, TaqMan<br>(3) cel-miR-39, miR-17	Yes	9	4–9 h	1	4–9 h: ≈90 fold<br>—	—	—	—	1.00	1.00	1.00
Wang, G.K. et al. 2010 [23]	miR-208a ↑	China<br>ED admission	33 AMI patients<br>Age: 63.5±10.1<br>Sex (m/f): 23/10	30 healthy adults<br>Age: 60.7±7.3<br>Sex (m/f): 20/10	Plasma	Biochemical markers (cardiac troponin I, cTnI >0.1 ng/mL), acute ischemic-type chest pain, electrocardiogram changes, and coronary angiography	(1) TRI Reagent BD<br>(2) qPCR, TaqMan<br>(3) cel-miR-39	No	8	4.8±3.5 h	Undetectable	<1 h	—	—	—	0.91	1.00	0.97
Wang, R. et al. 2011 [24]	miR-133 ↑<br>miR-328 ↑	China<br>Hospital admission	51 AMI patients<br>Age: 60.06±11.53<br>Sex (m/f): 43/8	28 healthy adults<br>Age: 57.86±10.36<br>Sex (m/f): 19/9	Plasma and whole blood	Chest pain lasting >20 min or diagnostic serial ECG changes consisting of new pathological Q waves or ST-segment and T-wave changes	(1) TRIzol LS<br>(2) qPCR<br>(3) Small nuclear RNA U6	No	7	5.24±1.38 h, 20 h, 7 days	ALL<br>—	ALL<br>—	miR-133<br>plasma: 4.4<br>w-blood: 4.4<br>miR-328<br>plasma: 10.9<br>w-blood: 16.1	ALL<br><24 h	1 w<br>1 w	0.98<br>—<br>—<br>—	0.73<br>—<br>—<br>—	0.89<br>0.702<br>0.81<br>0.872
Devaux, Y. et al. 2012 [25]	miR-208b ↑<br>miR-499↑	Luxembourg<br>ED admission	510 AMI patients<br>STEMI: n=397<br>Age: 62<br>Sex (m/f): 303/94<br>NSTEMI: n=113<br>Age: 62<br>Sex (m/f): 78/35	87 healthy adults<br>Age: 53<br>Sex (m/f): 87/0	Plasma	STEMI:<br>(*a*) clinically significant ST elevation (>1 mm); (*b*) completely occluded major coronary artery: TIMI, 0 flow in the left anterior descending, circumflex, or right coronary artery; (*c*) peak CK activity>600 U/L.<br>NSTEMI:<br>(*a*) no significant ST-elevation but clinically significant ST depression (>1 mm); (*b*) clinically significant lesion in a major coronary artery requiring PCI; (*c*) positive troponin concentrations after 24 h (>0.03 μg/L)	(1) mirVana PARIS kit, SYBR-green PCR kit<br>(2) qPCR, TaqMan<br>(3) 3 *C. elegans* spiked-in miRNAs	Yes	10	miR-208b and miR-499 were detectable as early as 1 h after the onset of chest pain in all patients and at <3 h, 3–6 h, 6–12 h;	ALL<br>—	1 h<br>1 h	5*10^5<br>3*10^5	6–12 h<br>6–12 h	>12 h<br>>12 h	0.79<br>0.95	1.00<br>1.00	0.90<br>0.97
Vogel, B. et al. 2013 [26]	miR-181c*↑<br>miR-1915 ↓<br>miR -380* ↓<br>miR -455 ↓<br>miR -566 ↓<br>miR -636 ↓<br>miR -7-1* ↓	Germany	18 AMI patients<br>Age: 63±17	21 healthy adults<br>Age: 60±13	Whole blood	Patients with suspected STEMI with chest pain and ST-segment elevation<br>bundle branch blockage (LBBB) were recruited	(1) Geniom RT analyzer<br>(2) qPCR	No	7	miRNA expression levels were measured at 0 h, 2 h, 4 h, 12 h, and 24 h after onset of symptoms	ALL<br>—	ALL<br>0h	(intensity: AMI/control)<br>≈4.5<br>≈0.35<br>≈0.48<br>≈0.4<br>≈0.05<br>≈0.3<br>≈0.3<br>≈0.6<br>≈0.4	1 h<br>4 h<br>1 h<br>12 h<br>0 h<br>2 h<br>2 h<br>0 h<br>2 h	ALL<br>24 h	ALL<br>—	ALL<br>—	miR-1254: 0.85<br>miR -380*: 0.87<br>miR -455: 0.82<br>miR -566: 0.86<br>miR -636: 0.84<br>miR -7-1*: 0.82<br>6 miRNAs: 0.88
Gidlof, O. et al. 2013 [37]	miR-1 ↑<br>miR-208b ↑<br>miR-499-5p↑	Sweden<br>Hospital admission	319 AMI patients (173with STEMI 146 with NSTEMI)<br>Age: 65±11.1<br>Sex (m/f): —	88 non-AMI people with angina and chest pain<br>Age: 65±11.1<br>Sex (m/f): —	Plasma	ST-elevation myocardial infarction (STEMI) diagnosis was based on ECG criteria;<br>non-STEMI (NSTEMI) on troponin levels together with clinical symptoms of ischemia.<br>Non-MI was defined as absence of troponin-elevation and absence of ST-elevation.	(1) TRIzol LS reagent<br>(2) qPCR<br>(3) miR-17	No	8	Samples were taken at admission, at 24, 48 and 72 hours.	ALL<br>—	ALL<br><24h	ALL<br>—	ALL<br>—	ALL<br>—	0.55<br>0.75<br>0.78	0.60<br>0.82<br>0.75	0.57<br>0.82<br>0.79
Li, C. J. et al. 2013 [38]	miR-1 ↑<br>miR-134 ↑<br>miR-186 ↑<br>miR-208 ↑<br>miR-223↑<br>miR-499↑	China<br>Hospital admission	117 AMI patients<br>Age: 62.70±11.40<br>Sex (m/f): 97/20	100 healthy adults (age and gender matched)<br>Age: 61.10±7.97<br>Sex (m/f): 81/19	Serum	The AMI patients were clinically diagnosed as Universal Definition of Myocardial Infarction.	(1) TRIzol LS reagent<br>(2) qPCR<br>(3) U6 and 5S rRNA	No	9	within 2h after hospitalization.	1<br>1<br>1<br>1<br>1<br>1	ALL<br><2h	2h: 1.55,<br>2.68<br>1.99<br>4.09<br>1.57<br>1.80	ALL<br>—	ALL<br>—	0.60<br>0.55<br>0.78<br>0.78<br>0.78<br>0.75	0.70<br>0.75<br>0.58<br>0.76<br>0.68<br>0.72	0.696<br>0.657<br>0.715<br>0.778<br>0.741<br>0.755
Li, Y. Q. et al. 2013 [39]	miR-1 ↑<br>miR-133a ↑<br>miR-208b ↑<br>miR-499↑	China<br>Hospital admission	67 AMI patients<br>Age: 63.84±11.17<br>Sex (m/f): 52/15	32 healthy adults<br>Age: 61.75±9.58<br>Sex (m/f): 22/10	Plasma	AMI was diagnosed in patients with increased cTnT or creatine kinase-MB (CK-MB) levels combined with chest pain lasting .30 minutes and electrocardiogram (ECG) findings such as new pathological Q waves or ST-segment elevation or depression. Patients were excluded if they had received intravenous thrombolytic or anticoagulant treatment before the initial blood samples were acquired.	(1) TRIzol LS reagent<br>(2) qPCR<br>(3) cel-miR-39	No	9	Venous blood samples (3 mL) were collected on within 12 h of the onset of symptoms (4.6±2.9 h) and on Day 14	1<br>1<br>1<br>1	<12h	<12h: ≈3<br>≈6<br>≈40<br>≈10	ALL<br>—	>14d<br><14d<br><14d<br><14d	0.78<br>0.88<br>0.82<br>0.80	0.85<br>0.96<br>1.00<br>0.94	0.8265<br>0.9468<br>0.8899<br>0.8841

The characteristics given here included study design, study patients, changes in miRNAs over time, and the sensitivity, specificity, and AUC.

— : None reported

≈: approximately

m/f: male to female
