# Supplementary material for: MiRNAs as Biomarkers of Myocardial Infarction: A Meta-Analysis
Source: PLoS One. 2014 Feb 12;9(2):e88566. doi: 10.1371/journal.pone.0088566 (PMC3922900; doi:10.1371/journal.pone.0088566)
Supplement: Table S2 — QUADAS studies of diagnostic test accuracy. (PDF) [file pone.0088566.s002.pdf]

Table S2 QUADAS studies of diagnostic test accuracy.

Criteria were taken from Whiting et al. and modified for this review (2003)

	Representative spectrum	Acceptable reference standard	Acceptable delay between tests	Partial verification avoided	Differential verification avoided	Incorporation avoided	Reference standard results blinded	Index test results blinded	Inclusion criteria defined	Characteristics described (age and sex)	Multivariate analysis performed	Confounders comparable at baseline, adjusted for analysis and study design
Adachi, T. et al. 2010 [11]	?	?	+	+	+	+	—	—	—	+	—	—
Ai, J. et al. 2010 [12]	?	+	?	+	+	+	—	—	+	+	—	—
Cheng, Y. et al. 2010 [13]	+	+	+	+	+	+	—	—	+	+	—	—
Corsten, M.F. et al. 2010 [14]	?	+	+	+	+	+	—	—	+	+	—	—
D'Alessandra, Y. et al. 2010 [15]	—	+	+	+	+	+	—	—	+	+	+	+
Gidlof, O. et al. 2011 [16]	—	+	+	+	+	+	—	—	+	+	—	—
Kuwabara, Y. et al. 2011 [17]	+	+	+	+	+	+	—	—	+	+	—	—
Long, G. et al. 2012 [18]	?	+	+	+	+	+	—	—	+	+	—	—
Long, G. et al. 2012 [19]	?	+	+	+	+	+	—	—	+	+	—	—
Meder B. et al. 2011 [20]	—	+	+	+	+	+	—	—	+	+	—	—
Oerlemans, M.I.F.J. et al. 2012 [21]	+	+	+	+	+	+	—	—	+	+	+	+
Olivieri, F. et al. 2012 [22]	—	+	+	+	+	+	—	—	+	+	+	+
Wang, GK. et al 2010 [23]	+	+	+	+	+	+	—	—	+	+	—	—
Wang, R. et al. 2011 [24]	?	+	+	+	+	+	—	—	+	+	—	—
Devaux, Y. et al. 2012 [25]	+	+	+	+	+	+	—	—	+	+	+	+
Vogel, B. et al. 2013 [26]	?	+	+	+	+	+	—	—	+	+	—	—
Gidlof, O. et al. 2013 [37]	+	+	+	+	+	+	—	—	+	+	—	—
Li, C. J. et al. 2013 [38]	+	+	+	+	+	+	—	—	+	+	—	+
Li, Y. Q. et al. 2013 [39]	+	+	+	+	+	+	—	—	+	+	—	+

+: Yes; —: No; ?: unclear
