# Supplementary material for: MiRNAs as Biomarkers of Myocardial Infarction: A Meta-Analysis
Source: PLoS One. 2014 Feb 12;9(2):e88566. doi: 10.1371/journal.pone.0088566 (PMC3922900; doi:10.1371/journal.pone.0088566)
Supplement: Appendix S1 — Methodology checklist: modified QUADAS for measuring diagnostic test accuracy. (DOC) [file pone.0088566.s003.doc]

**Appendix S1** Methodology checklist: modified QUADAS for measuring diagnostic test accuracy

The criteria were taken from Whiting et al. and were modified for this review (2003).

Are inclusion criteria defined? Yes No Unclear

Are characteristics such as age and sex described? Yes No Unclear

Was a multivariate analysis performed? Yes No Unclear

Are confounders comparable at baseline or adjusted for the analysis or study design? Yes No Unclear

Was the spectrum of participants representative of the patients who will receive the test in practice, such as patients entering the emergency services department with chest pain and suspected ACS? Yes No Unclear

Was the reference standard likely to classify the target condition correctly? For example, was it based on the universal definition of MI? Yes No Unclear

Was the period between performance of the reference standard and the index test short enough to show that the target condition did not change between the two tests? For example, were the two tests both conducted within the 12 hour time frame required for the reference standard? Yes No Unclear

Did the whole sample or a random selection of the sample receive verification using the reference standard? Yes No Unclear

Did participants receive the same reference standard regardless of the index test result? Yes No Unclear

Was the reference standard independent of the index test? For example, did the index test form part of the reference standard? Yes No Unclear

Were the index test results interpreted without knowledge of the results of the reference standard? Yes No Unclear

Were the reference standard results interpreted without knowledge of the results of the index test? Yes No Unclear

Were uninterpretable, indeterminate, and intermediate test results reported? Yes No Unclear

Were the same clinical data available when test results were interpreted as would be available when the test is used in practice? Yes No Unclear

Were withdrawals from the study explained? Yes No Unclear

These criteria were included in the quality assessment of the CTCA/ETT studies because there was a risk of uninterpretable

results from imaging in this review. This was not the case with the biomarker review.
